# *Xenopus *importin beta validates human importin beta as a cell cycle negative regulator

**DOI:** 10.1186/1471-2121-9-14

**Published:** 2008-03-22

**Authors:** Valerie A Delmar, Rene C Chan, Douglass J Forbes

**Affiliations:** 1 Section of Cell and Developmental Biology, Division of Biological Sciences 0347, University of California – San Diego, 9500 Gilman Drive, La Jolla, CA 92093-0347, USA; 2 Department of Developmental Biology, Stanford University, Stanford, CA 94305, USA

## Abstract

**Background:**

Human importin beta has been used in all *Xenopus laevis in vitro *nuclear assembly and spindle assembly studies. This disconnect between species raised the question for us as to whether importin beta was an authentic negative regulator of cell cycle events, or a dominant negative regulator due to a difference between the human and *Xenopus *importin beta sequences. No *Xenopus *importin beta gene was yet identified at the time of those studies. Thus, we first cloned, identified, and tested the *Xenopus *importin beta gene to address this important mechanistic difference. If human importin beta is an authentic negative regulator then we would expect human and *Xenopus *importin beta to have identical negative regulatory effects on nuclear membrane fusion and pore assembly. If human importin beta acts instead as a dominant negative mutant inhibitor, we should then see no inhibitory effect when we added the *Xenopus *homologue.

**Results:**

We found that *Xenopus *importin beta acts identically to its human counterpart. It negatively regulates both nuclear membrane fusion and pore assembly. Human importin beta inhibition was previously found to be reversible by Ran for mitotic spindle assembly and nuclear membrane fusion, but not nuclear pore assembly. During the present study, we observed that this differing reversibility varied depending on the presence or absence of a tag on importin beta. Indeed, when untagged importin beta, either human or *Xenopus*, was used, inhibition of nuclear pore assembly proved to be Ran-reversible.

**Conclusion:**

We conclude that importin beta, human or *Xenopus*, is an authentic negative regulator of nuclear assembly and, presumably, spindle assembly. A difference in the Ran sensitivity between tagged and untagged importin beta in pore assembly gives us mechanistic insight into nuclear pore formation.

## Background

Vertebrate nuclear assembly is a complex process involving the sequential recruitment of specific proteins and membranes to chromatin. At the end of mitosis, membrane vesicles and/or ER membrane sheets arrive at the chromatin surface to fuse and form a unique structure consisting of two complete, encircling membrane bilayers [1,2]. As soon as regions of double membrane form at the chromatin surface, nuclear pore complexes form within those regions perforating the membranes. Nuclear pore complexes span the bilayers and control virtually all traffic between the nucleus and cytoplasm [3,4]. The 125-megadalton vertebrate nuclear pore is composed of multiple copies of ~30 different nucleoporins, only three of which are integral membrane proteins [5]. The majority of nucleoporins are recruited from soluble cytoplasmic subunits. The assembly of these nucleoporins into the 500–1000 protein complex is a daunting task, as nucleoporins must sequentially and precisely assemble in the correct order and location [6-8]. Determining the choreographed molecular mechanism by which nucleoporins assemble into functional pores within the double nuclear membranes is a matter of intense research.

The nuclear import factor, importin beta, and its regulatory counterpart, the small GTPase Ran, were revealed to be two key regulatory factors controlling this choreography, both for nuclear membrane fusion and separately for nuclear pore assembly [9-13]. Addition of excess human importin beta to a *Xenopus *nuclear reconstitution system disrupts the endogenous ratio between importin beta and RanGTP. This disruption blocks proper nuclear membrane fusion and the subsequent step of nuclear pore assembly [9,10]. The block to nuclear membrane fusion was found to be reversible by the positive regulator, RanGTP, but the block to pore assembly, oddly, was not [9,10]. There is, however, much precedence for positive Ran effects on nuclear pore assembly: The addition of RanQ69L, a Ran mutant constitutively in the GTP-bound state, to the *Xenopus *reconstitution system causes greatly increased nuclear pore assembly and ectopic formation of additional pores in cytoplasmic membranes or annulate lamellae [9,10,14-17]. These studies led to the hypothesis that importin beta acts in the cell cycle to negatively regulate nuclear pore formation and that it does so by binding to nucleoporins, preventing them from interacting with one another. When such importin beta/nucleoporin complexes enter the vicinity of high RanGTP, importin beta preferentially binds RanGTP, releasing its hold on the nucleoporins. A high concentration of RanGTP is produced only around chromatin, due to the chromosomal localization of the RanGEF, RCC1 [18-21]. The freed nucleoporins are then able to interact with one another in the correct location and the correct ratio to form nuclear pores at the chromatin periphery [9,10,22].

Prior to the discovery of its role as a negative regulator of nuclear membrane fusion and pore assembly, importin beta was elegantly shown by a number of groups to be a negative regulator of mitotic spindle assembly in *Xenopus laevis *egg extract [23-29], mammalian cell lines [25,30], *Drosophila Melanogaster *[31], and *Caenorhabditis elegans *[32] (Reviewed in [11,12,33,34]). In this arena, mitotic spindle assembly factors (SAFs) such as TPX2, NuMa, and XCTK2 are found to be imported into the nucleus by importin beta and localize there throughout interphase in Xenopus egg extract [27,28,35-37] and mammalian cell lines [35,38] (Reviewed in [39-41]). This sequestration effectively prevents the SAFs from interfering with interphase microtubule formation in the cytoplasm. At mitosis when the nuclear envelope breaks down, the SAFs are released from the nucleus and come under importin beta regulation. Binding of importin beta inhibits the SAFs throughout the cell, except in the vicinity of the RanGTP-rich chromosomes. There, importin beta preferentially binds to RanGTP, releasing its hold on the spindle assembly factors and allowing them to initiate mitotic spindle formation around the chromosomes.

These nuclear and spindle assembly studies on the regulatory role of importin beta were performed in interphase and mitotic assembly systems derived from *Xenopus *eggs [23,26-28,35,42-50]. In a *Xenopus *interphase egg extract, nuclei normally assemble spontaneously around added chromatin or DNA [51-60]. In contrast, in a *Xenopus *mitotic egg extract, spindles spontaneously form around the added chromatin [61,62]. Thus, these *in vitro *systems are powerful tools for studying both nuclear and mitotic spindle assembly.

Upon further analysis, we realized that the recombinant importin beta used in all the *Xenopus *studies of nuclear and spindle assembly was, in actuality, *human *importin beta [9,10,25,27-30,37,63-68]. (*Xenopus *importin beta had neither been identified nor cloned and thus was not available for the studies). The use of recombinant human importin beta in the *Xenopus *system led to a further key question: Is importin beta an *authentic *negative regulator of cellular function, or does human importin beta act as a dominant negative mutant as a result of sequence variation between the human and *Xenopus *proteins?

To address this question, in this study we identified, cloned, and tested recombinant *Xenopus *importin beta for its role in nuclear membrane fusion and nuclear pore assembly. We found *Xenopus *importin beta to act identically to human importin beta, i.e., it acts as a negative regulator of both nuclear membrane fusion and pore assembly, finally validating the conclusion that importin beta is an authentic negative regulator of cell cycle steps. However, in examining tagged importin betas, which include the form that has been used in all the previous studies, we found evidence that the tag renders importin beta mutant in its response to Ran, but does so specifically with respect to pore assembly. This impairment of importin beta raises interesting hypotheses as to why nuclear pore assembly is unique, which will be discussed here.

## Results

### Identification and cloning of *Xenopus laevis *importin beta

**Figure 1:**
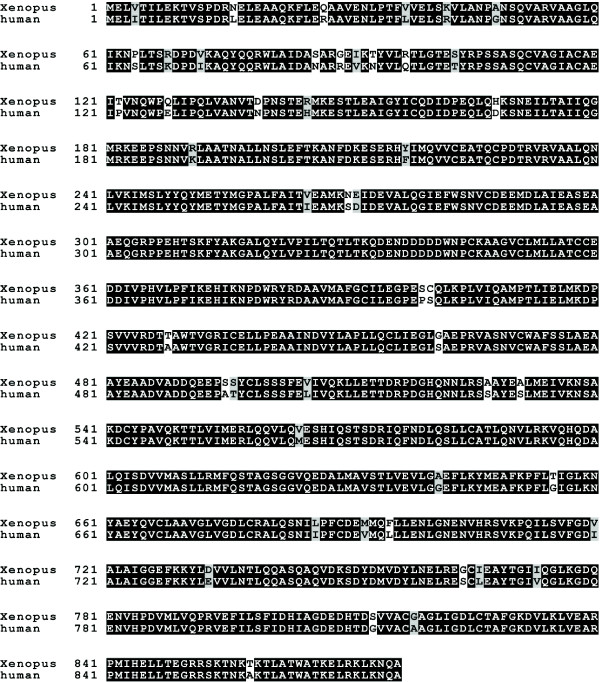
***Xenopus *importin beta shows close homology to human importin beta**. The protein sequence of *Xenopus *importin beta shows very close homology to human importin beta with 94% identities (828/876, black boxes) and 97% positives (857/876 gray and black boxes). The amino acid composition, along with the length of the protein, is well conserved between *Xenopus *and human importin beta. Three of the conservative amino acid differences between the *Xenopus *and human importin beta sequence are at residues involved in FG-domain binding (F217Y [82–84], I265V [84], and L505V [84]).

To address whether human importin beta acts as an authentic negative regulator of nuclear membrane fusion, pore assembly, and spindle assembly, or as a dominant negative mutant inhibitor due to inherent species sequence differences, we set out to identify and clone *Xenopus *importin beta. Overlapping *Xenopus *EST sequences showing homology to human importin beta were compiled from gene fragments present in the *Xenopus *EST database. A full-length *Xenopus *importin beta sequence was then cloned from total *Xenopus *RNA by reverse transcription and PCR. The resulting full-length *Xenopus *importin beta cDNA was cloned into an N-terminal His tag vector, pET28a, for both protein expression and sequencing. The corresponding nucleotide sequence was submitted to GenBank, Accession number EU286786. Sequence alignment revealed that *Xenopus *importin beta is 94% identical to human importin beta; however, 48 amino acids varied between the species, although often in a conserved manner (Figure 1). These 48 amino acids give scope for the hypothesis that potential "mutant" amino acids could cause a dominant negative phenotype with human importin beta.

To further eliminate any potential differences from endogenous *Xenopus *importin beta, we wished to use recombinant *Xenopus *importin beta free of purification tags. For this, the *Xenopus *importin beta clone was subcloned into a vector that introduced a cleavable GST tag. After the GST- importin beta was expressed and purified, the GST tag was removed by Precision Protease and the resulting untagged *Xenopus *protein was used in nuclear assembly studies.

### *Xenopus *importin beta negatively regulates membrane fusion in a Ran-sensitive manner

**Figure 2:**
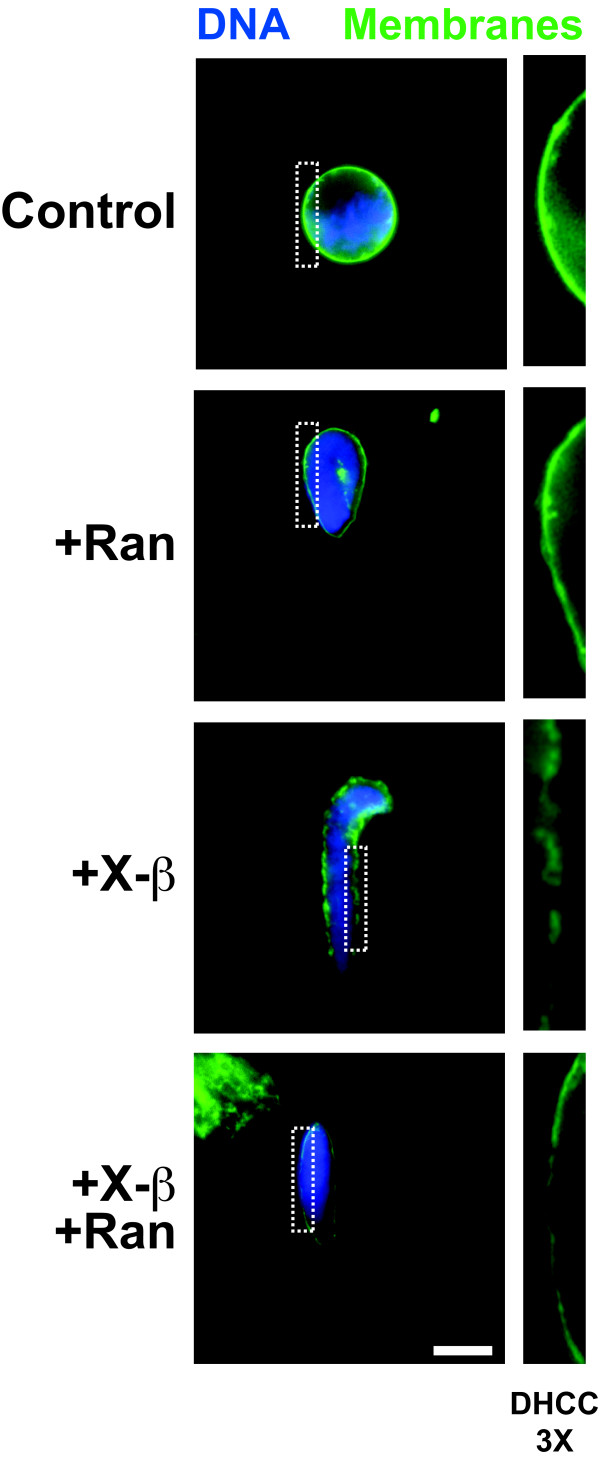
***Xenopus *importin beta is an authentic negative regulator of the fusion events in nuclear membrane formation**. Addition of His-tagged *Xenopus *importin beta to a nuclear assembly reaction (+X-β) blocked nuclear membrane fusion, as shown by the lack of a solid nuclear rim stain by the green fluorescent membrane dye DHCC. The block to membrane fusion could be rescued by the addition of RanQ69L-GTP (+X-β +Ran). Where indicated, the added concentrations were 30 μM *Xenopus *importin beta and/or 40 μM RanQ69L-GTP. DNA was stained with DAPI. These observations are in accordance with experiments done with recombinant human importin beta in nuclear assembly reactions [9]. To better view the membranes, a section of the membrane stain (white dashed box) is enlarged by 3X (right panels). The bar represents 10 microns.

With the *Xenopus *importin beta clone in hand, we set out first to ask whether it blocked nuclear membrane fusion when in excess. If no importin beta is added to a *Xenopus laevis in vitro *system, after one hour smooth fused membranes are formed and can be visualized with the membrane dye DHCC, as we also observed here (Figure 2, Control) [9,69]. However, when we added excess untagged *Xenopus *importin beta at the beginning of a nuclear reconstitution reaction, nuclear membrane formation was blocked, as shown by the presence of fuzzy unfused membranes (Figure 2, +X-β). This inhibition of fusion was reversed by addition of RanQ69L-GTP, a form of Ran stably associated with GTP, as it cannot hydrolyze GTP (Figure 2, +X-β + Ran) [16]. These results thus indicated that *Xenopus *importin beta acts identically to human importin beta in negatively regulating nuclear membrane fusion, and does so in a Ran-sensitive manner.

### *Xenopus *importin beta negatively regulates nuclear pore assembly and is reversed by Ran

**Figure 3:**
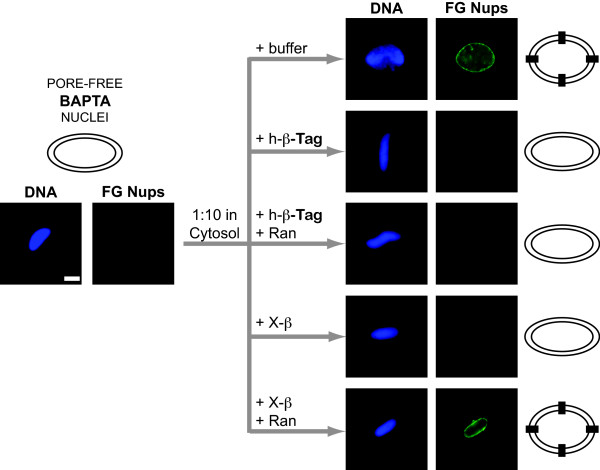
***Xenopus *importin beta is an authentic negative regulator of nuclear pore assembly and is reversed by RanGTP**. Pore-free BAPTA nuclear intermediates, which have fused nuclear membranes but contain no nuclear pores (left panel), when diluted into fresh cytosol (+ buffer), incorporate nuclear pores. The addition of His-tagged human importin beta (+h-β-Tag) or *Xenopus *untagged importin beta (+X-β) prevented nuclear pore assembly. Addition of RanQ69L-GTP with His-tagged human importin beta (+h-β-Tag +Ran) could not reverse the beta block to pore assembly, as previously observed [9]. However, addition of RanQ69L-GTP with untagged *Xenopus *importin beta (+X-β +Ran) did reverse the beta block to pore assembly. Nuclear pores were detected by the monoclonal antibody mAb414, which recognizes FG nucleoporins (FG Nups). Where indicated, importin beta was added at 20 μM and RanQ69L-GTP at 30 μM. The bar represents 10 microns. Black squares on the drawings at the right indicate FG-staining nuclear pores.

We next tested *Xenopus *importin beta for inhibition of nuclear pore assembly. We had previously shown that human importin beta blocks nuclear pore formation, but cannot be reversed by Ran [9]. To investigate the effect of *Xenopus *importin beta on pore assembly, we first needed to bypass the inhibition of nuclear membrane fusion and look only at the nuclear pore assembly step. It has long been known that when the Ca^++ ^chelator BAPTA is added to a *Xenopus *nuclear reconstitution reaction at t = 0', nuclei result that have a fused nuclear envelope, but no nuclear pores [9,58,70]. These "BAPTA pore-free nuclei," in consequence, do not stain with antibody directed against nucleoporins containing Phenylalanine-Glycine (FG) repeats (Figure 3, left panels) [9,58]. Upon dilution of the BAPTA nuclei into *Xenopus *cytosol free of BAPTA, nuclear pores form normally, as previously described and shown here (Figure 3, cytosol + buffer) [9]. This ability of BAPTA pore-free nuclei to be rescued provides a convenient system for investigating solely the effect of *Xenopus *importin beta on pore assembly [58]. Here we found that, when BAPTA nuclei were diluted into cytosol containing *Xenopus *importin beta, the nuclei were not able to form nuclear pores (Figure 3, +X-β), identical to the block seen with human importin beta [9]. Thus, we conclude that importin beta, either *Xenopus *or human, is indeed an authentic negative regulator of nuclear pore assembly.

Strikingly, when BAPTA nuclei were diluted into cytosol containing *Xenopus *importin beta and RanQ69L, the BAPTA defect was rescued by Ran, i.e., FG-containing nuclear pores formed (Figure 3, bottom panel, +X-β +Ran). This rescue differed from what was previously seen where Ran was unable to overcome the human importin beta block to pore assembly (see Figure 3, + h-β-Tag +Ran and [9]). This new result prompted us to investigate the cause for the unexpected difference in Ran sensitivity.

### Tagging importin beta causes insensitivity to Ran in its block to nuclear pore assembly

**Figure 4:**
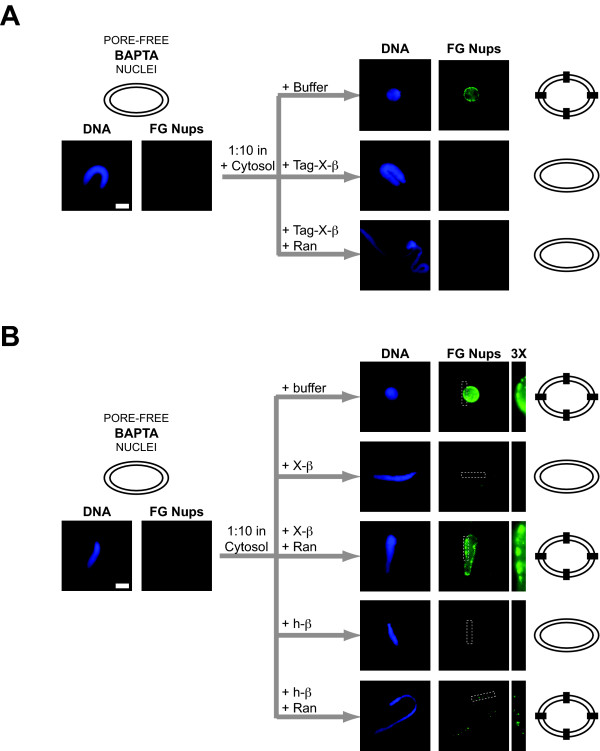
**Altering importin beta by addition of a His-tag renders importin beta insensitive to RanGTP specifically in its block to nuclear pore assembly**. **A. **Pore-free BAPTA intermediates rescued in the presence of cytosol plus His-tagged *Xenopus *importin beta were not able to assemble nuclear pores (+Tag-X-β). When RanQ69L-GTP was added along with His-tagged *Xenopus *importin beta, the block to pore assembly could not be reversed (+Tag-X-β +Ran). Where indicated, importin beta was added at 10 μM and RanQ69L-GTP at 50 μM. The bar represents 10 microns. **B. **Pore-free BAPTA nuclear intermediates rescued in the presence of cytosol and untagged human or *Xenopus *importin beta were not able to assemble nuclear pores (+X-β or +h-β). The inhibitory concentration of 10 μM used here was determined to be the approximate minimum concentration for pore assembly inhibition in a separate experiment (data not shown). When RanQ69L-GTP was added along with untagged human importin beta, the block to pore assembly was partially reversed (+h-β +Ran). The *Xenopus *importin beta block was fully reversed (+X-β +Ran). To better visualize the FG-nucleoporin stain, a section of the images (white dashed box) was enlarged by 3X (right most panel). Where indicated, importin beta was added at 10 μM and RanQ69L-GTP at 50 μM. The bar represents 10 microns.

We considered the differing Ran reversibility results seen with human and Xenopus importin beta. Two possibilities existed: 1) either human importin beta differs from *Xenopus *importin beta with respect to its sensitivity to Ran, because of an inherent sequence difference in the importin beta coding sequence, or, 2) the His-tag present on the human importin beta used in all previous *in vitro *studies alters its sensitivity to Ran in a detrimental manner, but only with respect to pore assembly. To distinguish between these two mechanistic explanations, the BAPTA rescue experiment was next performed using *tagged *Xenopus importin beta, where an N-terminal His-tag was introduced. We found that tagged *Xenopus *importin beta acted identically to tagged human beta, i.e., it was not reversible by Ran (Figure 4A). Thus, the second model of tag-induced insensitivity to Ran appeared correct.

As a final test, however, an untagged form of human importin beta was cloned and used in a rescue experiment. We found that untagged human importin beta blocked the ability of nuclear pores to form when BAPTA-arrested nuclei were diluted into fresh cytosol (Figure 4B, +h-β). However, now RanQ69L rescued the pore assembly defect, albeit not as strongly as with the untagged *Xenopus *importin beta homologue (Figure 4B, compare +h-β +Ran with +X-β + Ran). Therefore, the first model of human importin beta acting as a dominant negative due to sequence variation is also plausible. Taken together, the data indicate that, specifically with respect to importin beta's block to pore assembly, wild-type human importin beta is less sensitive to Ran than *Xenopus *importin beta, and the presence of a His-tag on human importin beta renders it insensitive to Ran.

## Discussion

In this study we validate importin beta as a negative regulator of cell cycle events, including nuclear membrane fusion and pore assembly. As all importin beta studies on nuclear and mitotic spindle formation using the *Xenopus in vitro *system to date have involved the addition of human importin beta, we asked whether the effects of importin beta were due to an inter-species sequence variation causing the human protein to act as a dominant negative *mutant *form. Instead we clearly show in experiments with *Xenopus *importin beta that this wild type protein acts as a true negative regulator.

Interestingly, during the course of this study we uncovered a mechanistic explanation for the Ran-insensitive importin beta block to pore assembly previously observed [9]. Tagging importin beta at the N- (*Xenopus*) or C- (human) terminus was discovered to block importin beta's sensitivity to RanGTP (up to 100 μM of added Ran, data not shown) in *Xenopus in vitro *studies, but only in the realm of nuclear pore assembly. Both spindle assembly and nuclear membrane assembly are blocked by importin beta, but readily reversed by RanGTP [9]. We showed that, upon removal of the tag, RanGTP now also reversed the block to pore assembly engendered by *Xenopus *importin beta and partially reverses the block by human importin beta.

Importin beta normally undergoes a significant conformational change upon RanGTP binding [71-80]. It is therefore not inconceivable that even a small tag, such as the six histidine tag, could increase rigidity or cause an inability for importin beta to fully change conformation and thus be unable to release its binding partners correctly in response to RanGTP. What is surprising is that the tagged-importin beta insensitivity to RanGTP is only seen with respect to its role as a negative regulator of nuclear pore assembly. All other studies on the dynamics of importin beta and RanGTP in mitotic spindle assembly and nuclear membrane fusion have not shown an unresponsiveness of tagged-importin beta to RanGTP [9,10]. One explanation for this might derive from the known association of importin beta with multiple FG-nucleoporins, suggesting that multiple sequential steps in pore assembly could potentially be regulated by importin beta [74,81-84]. The cumulative effect of an impaired importin beta being incompletely released by Ran at *each *step of pore assembly could explain the observed irreversibility of tagged importin beta's block specifically on nuclear pore assembly.

A second explanation for why importin beta's regulation of nuclear pore complex assembly differs from nuclear membrane fusion and spindle assembly with respect to Ran reversibility may involve how the targets of regulation interact with importin beta. What mechanistically might differ between spindle assembly factor (SAF) binding and nucleoporin (Nup) binding to importin beta? One study suggested a region of importin beta (aa 71–876) bound to SAFs and blocked spindle assembly when added to a mitotic extract, whereas amino acids 1–380 of importin beta had a lesser effect on spindle assembly [27], albeit other interpretations are also possible [38]. Notably, importin beta has two known binding sites for nucleoporins, aa 1–396 near the N-terminus and aa 304–876 near the C-terminus [83]. Importantly, the N-terminal Nup binding site of importin beta partially overlaps with the binding site for RanGTP [12,72,73,82,83,85,86]. An intriguing possibility is that this N-terminal Nup binding site could be responsible for tagged importin beta's insensitivity to RanGTP with respect to pore assembly, as this site appears not to play a significant role in the regulation of mitotic spindle assembly.

There are as yet no identified molecular targets of importin beta with respect to nuclear membrane fusion that can be similarly analyzed. However, when an importin beta fragment (aa 45–462) containing the N-terminal Nup binding site, but lacking the importin alpha, RanGTP, and C-terminal Nup binding sites, is added, nuclear membrane fusion goes forward [9]. Thus, the binding site on importin beta for the unknown membrane fusion factor or factors is not contained within this region (aa 45–462).

Perhaps the most surprising difference between tagged and untagged importin beta sensitivity to Ran is the differing effect on annulate lamellae (AL) pore formation versus nuclear pore formation. Importin beta blocks AL formation, but this block is reversed by RanGTP, whether tagged or untagged importin beta is used ([10] and data not shown), which is clearly not the case for nuclear pore assembly. One explanation could be that AL formation may not be as stringent as nuclear pore assembly, as the pore complexes in AL do not necessarily need to function, whereas nuclear pore complexes must be functional. An alternative explanation could be that the tagged importin beta blocks an assembly step that is unique to *nuclear *pore assembly and not found in AL assembly. Whatever the tag-sensitive block to nuclear pore assembly is, it must occur *after *nuclear vesicle-vesicle fusion, as the importin beta block to pore assembly is observed using membrane-enclosed BAPTA intermediates as a starting point (Figures 3 and 4) [9].

The placement of the 6-Histidine tag at either the N- or C-terminus of importin beta appears not to matter. The human importin beta used in most *Xenopus in vitro *studies [9,10,26,29,30,35,63,65,87] has a His tag at its C-terminus, while the tagged *Xenopus *importin beta constructed in this study has the tag at the N-terminus. We have not tested other types of tags on importin beta for their effect on pore assembly. Clearly, in the future functional studies using importin beta should take care to use an untagged version of importin beta or, alternatively, may specifically want to use a tagged version in order to study the mechanism of arrested nuclear pore assembly more closely.

## Conclusion

By using species-specific importin beta for nuclear assembly studies we have now demonstrated that importin beta, human or *Xenopus*, is indeed an authentic negative regulator of nuclear assembly and, presumably, spindle assembly. In previous studies, the action of human importin beta could easily have been due to a dominant negative mutant effect, which would have required a different model of regulation. By performing the experiments here we now provide the evidence that importin beta must truly be a negative regulator in its wild type form.

## Methods

### Cloning and Sequencing of *Xenopus *importin beta

To obtain a sequence of *Xenopus *importin beta, overlapping *Xenopus *EST sequences showing homology to human importin beta were compiled from fragments present in the NIH *Xenopus *EST database. Full-length *Xenopus *importin beta was then cloned from *Xenopus *total RNA by reverse transcription and polymerase chain reaction (PCR) amplification using the forward primer 5'-CCCGGATCCATGGAGCTCGTCACCATCCTC-3' (with BamHI site underlined) and reverse primer 5'-CCCCGCGGCCGCTCAGGCTTGGTTTTTCAG-3' (with NotI site underlined). The full-length *Xenopus *importin beta cDNA was cloned into the N-terminal His tag vector pET28a (Invitrogen, Carlsbad, CA) (pET28a-Xbfl). GST-*Xenopus *importin beta (pGEX6P-Xbfl) was cloned by restriction digestion of pET28a-Xbfl with BamHI and NotI, and ligation of the insert into the pGEX6P-3 vector (Amersham Biosciences, Sweden) digested with the same restriction enzymes.

The sequence of *Xenopus *importin beta was confirmed by DNA sequencing of the pET28-Xbfl construct with two forward primers: T7 promoter and an internal primer (Xbfl intF1, 5' GCTGCACTGCAAAACCTGG 3') and a reverse primer, the T7 terminator primer. Human and *Xenopus *importin beta were aligned using the Clustal-W program and highlighted using BoxShade, both available through the Workbench program of the San Diego Super Computer Center [88].

### Protein Expression and Purification

His-tagged proteins (*Xenopus *importin beta, human importin beta, and RanQ69L), were expressed and purified as previously described [9]. RanQ69L was loaded with GTP as described previously [9].

To purify untagged human and *Xenopus *importin beta, pGEX6P-hbfl and pGEX6P-Xbfl were transformed into Rosetta DE3 competent cells (EMD Biosciences, Germany), expanded, and induced with 0.1 mM isopropyl-beta-D-thiogalactopyranoside (IPTG) overnight at 17°C. Glutathione-Sepharose 4B beads (Amersham Biosciences, Sweden) were used to purify the GST-tagged protein as per manufacturer's instructions. To remove the GST tag, purified proteins were cleaved on the column in the presence of 80 units of Precision Protease (Amersham Biosciences, Sweden) for 4 hours at 4°C. Untagged protein was eluted from the column and dialyzed into 5% glycerol/PBS and stored at -80°C.

### Nuclear reconstitution and immunofluorescence

Nuclear reconstitution and 1,2-bis (2-aminophenoxy) ethane-N,N,N,N-tetraacetic acid (BAPTA) (Calbiochem, La Jolla, CA) nuclear reconstitution reactions were performed in the *Xenopus *egg extract system as described previously [9]. FG nucleoporins were localized using an Alexa-488 directly labelled monoclonal antibody mAb414 (Covance, Berkeley, CA). *Xenopus *egg cytosol and membranes were prepared as previously described [56], except for the use of 500 mM KCl in the membrane wash buffer. After fixation in 3% formaldehyde, membranes were visualized by the lipophilic dye 3,3-dihexyloxacarbocyanine iodide (DHCC) (Eastman Kodak, Rochester, NY). DNA was stained with 4',6-diamidino-2-phylindole (DAPI). Nuclei were visualized with an Axioskop 2 microscope (63X objective; Carl Zeiss, Thornwood, NY).

## Authors' contributions

VAD carried out the comparison between human and *Xenopus *importin beta and tagged vs. untagged importin beta. VAD drafted the manuscript.

RCC conceived of the original project, drafted parts of the manuscript, directed the cloning and sequencing of *Xenopus *importin beta, and performed preliminary characterization of His-tagged *Xenopus *importin beta.

DJF significantly contributed to the intellectual content and manuscript.

All authors read and approved the final manuscript.
